# Predicting 1-year mortality after hospitalization for community-acquired pneumonia

**DOI:** 10.1371/journal.pone.0192750

**Published:** 2018-02-14

**Authors:** Ane Uranga, Jose M. Quintana, Urko Aguirre, Amaia Artaraz, Rosa Diez, Silvia Pascual, Aitor Ballaz, Pedro P. España

**Affiliations:** 1 Department of Respiratory Medicine, Galdakao-Usansolo Hospital, Galdakao, Bizkaia, Spain; 2 Research Unit, Galdakao-Usansolo Hospital - Health Services Research on Chronic Patients Network (REDISSEC), Galdakao, Bizkaia, Spain; Kliniken der Stadt Köln gGmbH, GERMANY

## Abstract

**Background:**

Community-acquired pneumonia (CAP) is a major public health problem with high short- and long-term mortality. The main aim of this study was to develop and validate a specific prognostic index for one-year mortality in patients admitted for CAP.

**Methods:**

This was an observational, prospective study of adults aged ≥18 years admitted to Galdakao-Usansolo Hospital (Bizkaia, Spain) from January 2001 to July 2009 with a diagnosis of CAP surviving the first 15 days. The entire cohort was divided into two parts, in order to develop a one-year mortality predictive model in the derivation cohort, before validation using the second cohort.

**Results:**

A total of 2351 patients were included and divided into a derivation and a validation cohort. After deaths within 15 days were excluded, one-year mortality was 10.63%. A predictive model was created in order to predict one-year mortality, with a weighted score that included: aged over 80 years (4 points), congestive heart failure (2 points), dementia (6 points), respiratory rate ≥30 breaths per minute (2 points) and blood urea nitrogen >30 mg/dL (3 points) as predictors of higher risk with C-index of 0.76. This new model showed better predictive ability than current risk scores, PSI, CURB65 and SCAP with C-index of 0.73, 0.69 and 0.70, respectively.

**Conclusions:**

An easy-to-use score, called the one-year CAPSI, may be useful for identifying patients with a high probability of dying after an episode of CAP.

## Introduction

Community-acquired pneumonia (CAP) is a major public health problem with high morbidity and mortality [1]. The annual incidence ranges between two and eight cases per thousand inhabitants [2]—together with influenza, it was the seventh cause of mortality in the United States in 2008 [3]. Short-term mortality rates are high in patients admitted for CAP, but are even higher in those admitted to intensive care units, and up to 50% if there is a need for vasopressors [4]. Long-term mortality also remains high, with rates of 8%, 21%, and 36% within 90 days, one year, and five years, respectively [5].

An acute condition in older adults that requires hospitalization often implies a subsequent clinical deterioration. Recently, the need for hospital admission was related to a higher one-year mortality rate in patients admitted for any reason [6]. However, the relationship between pneumonia and long-term mortality is controversial. Patients admitted for pneumonia have exhibited higher long-term mortality rates than those admitted for other reasons [7,8]. In addition, various studies have tried to identify predictive factors for long-term mortality. Age has been postulated as one of the main predictors of mortality. The number of individuals aged over 65 years has increased in recent years, with that number expected to rise from 12% in 2000 to 20% in 2030, perhaps even doubling in 2050 [9]. Overall, the older adult population suffers from a greater number of comorbidities, and functional status is often poor. Other risk factors, such as male sex, race or patients suffering from healthcare-associated pneumonia (HCAP) are also related to increased mortality [1].

Despite current evidence regarding risk factors for one-year mortality after CAP, predicting long-term prognosis after an episode remains challenging. To date, no risk scores have been developed for predicting one-year mortality in hospitalized patients with CAP. The main aim of this study was to create and validate a prognostic index for one-year mortality in patients admitted after a CAP episode.

## Materials and methods

### Study design

This was an observational, prospective study of adults aged ≥18 years hospitalized with CAP surviving the first 15 days from January 2001 to July 2009. All participants provided written or verbal informed consent depending on the phase of the study, before their inclusion in the study and after being informed and having discussed its goals, risks, and potential benefits. The study was approved by the Ethics Research Committee of Hospital of Galdakao-Usansolo.

### Setting and study population

The study was carried out in Galdakao-Usansolo Hospital, a 400-bed teaching hospital in the Basque Country (northern Spain) that serves a population of 300 000 inhabitants. This medical institution belongs to the network of public hospitals of the Basque Healthcare Service, which provides free unrestricted healthcare to nearly 100% of the population. Hospitalized patients diagnosed with CAP were recruited from January 2001 to July 2009. Eligible patients were ≥18 years old, hospitalized with a diagnosis of CAP. Pneumonia was defined as pulmonary infiltrate on chest X-ray not seen previously, plus at least one symptom compatible with pneumonia such as cough, fever, dyspnoea, and/or chest pain [10]. Patients were excluded if they died within the first 15 days of diagnosis, had been discharged from an acute care hospital, an onsite subacute care unit, or a palliative care unit within the previous 14 days, or were HIV positive or chronically immunosuppressed (defined as immunosuppression for solid organ transplantation, having undergone a splenectomy, receiving ≥10 mg/d of prednisone or the equivalent for >30 days, taking other immunosuppressive agents, or having neutropenia, i.e. neutrophil count <1000/μL).

### Data collection

Demographic and clinical data for each patient were collected at baseline from medical records, and included comorbidities, physical examination, radiological presentation, and laboratory tests, as well as complications during admission. Only complications before 15 days were included in the model. Antibiotic treatment was assessed according to Spanish Society of Pulmonology and Thoracic Surgery (SEPAR) guidelines [11]. Disease severity was determined using the PSI (pneumonia severity index), CURB65 (confusion, urea, respiratory rate, blood pressure, age >65), and SCAP (severe community-acquired pneumonia) scores, calculated within the first 24 hours after diagnosis [10,12,13]. All patients were managed according to clinical guidelines that guaranteed the prospective and systematic collection of relevant clinical information. The TRIPOD statement relating to clinical prediction rules was adhered to in all cases [14]. One-year mortality was retrospectively assessed using the Basque Healthcare Service computer system.

### Assessment of outcomes

Primary outcome was one-year mortality after admission for CAP. Patients who died within the first 15 days after diagnosis were excluded in order to avoid the impact of severity of illness on mortality.

### Statistical analyses

Descriptive statistics included frequency tables and means and standard deviation (SD), or medians and interquartile ranges (IQRs). No assumptions were made in relation to missing values. The entire cohort was split randomly into derivation (50%) and validation (50%) sets. To assess the homogeneity of both samples, categorical variables were compared using Chi-squared and Fisher’s exact tests, and continuous variables with Student’s t-tests or non-parametric Wilcoxon tests. Univariate analysis using the Chi-squared/Fisher’s exact test (categorical variables) or t-test (continuous effects) was performed in the derivation cohort to identify the variables related to one-year mortality. Variables with statistically significant results at p <0.20 were entered into the multivariate model but variables retained in the final model have to be p<0.05.

Firstly, a multivariate survival model was developed with the selected variables using the backward elimination procedure. In order to obtain a robust model, age was dichotomized into 2 groups: <80 and ≥80. Moreover, an algorithm used to categorize variables was applied (CatPredi package) to ensure that this classification was the optimal. The hazard ratios (HR) and 95% confidence intervals (CI) of all selected variables were provided. The beta coefficients from the survival model were used to weight the relative importance of each variable for calculation of the prediction score. For weights, the beta coefficient for each predictor variable in the model was divided by the variable with the lowest beta coefficient and rounded to the nearest whole number. This produced a “relative weight” of each variable in relation to its ability to predict each outcome. To test its validity, the prognostic index (PI) was computed in the validation set as reported for the derivation cohort. After that, we developed a Cox model in the validation set, considering the PI as the covariate to estimate its beta regression coefficient (slope). If the beta regression coefficient of the slope is 1, the model is valid. Moreover, a likelihood-ratio test was performed to contrast that the slope is 1. The weights of the variables for each patient were then added together to produce the prediction scores for each patient, and three categories were established (mild, moderate and severe based on predicted versus observed one-year mortality. Risk categories were replicated in the validation cohort. Next, the predictive accuracy of the different risk scores was assessed in the derivation and validation cohorts using the C-index. Akaike information criteria (AIC) and R-squared (R^2^) were provided in both cases. The Greenwood-Nam D’Agostino (GND) method was used to assess the ability of the model to match predicted and observed one-year mortality rates in all developed survival models. Finally, Kaplan-Meier survival curves were used to assess operating severity scores for one-year mortality.

All effects were considered significant at p<0.05, unless otherwise stated. All statistical analysis was performed using SAS for Windows, version 9.4 (SAS Institute, Cary, NC). Figures were prepared using R version 3.3.0.

## Results

A total of 2351 patients, with 1208 and 1143 in the derivation and validation cohorts, respectively, were included. Baseline characteristics are shown in S1 Table. The mean age (SD) of the entire cohort was 69 (16.58) years, with 784 (33.35%) patients ≥80 years old. Both cohorts were similar except for the chronic obstructive pulmonary disease (COPD) rate, which was higher in the validation cohort. In total, 208 (7.99%) patients died during hospitalization, while 251 patients died within 15 days of diagnosis. After deaths within 15 days were excluded, one-year mortality was 10.84% (131 patients) and 10.41% (119 patients) in the derivation and validation cohorts, respectively (p = 0.74). Table 1 shows baseline characteristics among survivors and non-survivors at one-year.

**Table 1:** Baseline characteristics of survivors and non-survivors at one-year in the derivation cohort.

	Alive (n = 1077)	Dead (n = 131)	p-value
**Age, mean (SD)**	68.46 (16.71)	79.71 (10.23)	<0.0001
**Sex, n (%)**			0.44
Male	698 (64.81%)	90 (68.70%)	
Female	379 (35.19%)	41 (31.30%)	
**Alcohol consumption (yes), n (%)**	58 (5.40%)	5 (3.88%)	0.67
**Comorbidities, n (%)**			
Diabetes mellitus	166 (15.50%)	25 (19.38%)	0.25
COPD	249 (23.18%)	29 (22.48%)	0.91
Cancer	59 (5.48%)	17 (12.98%)	0.0033
CHF	65 (6.04%)	16 (12.21%)	0.01
CAD	101 (9.40%)	18 (13.95%)	0.12
CVD	80 (7.43%)	25 (19.08%)	<0.0001
Dementia	81 (7.53%)	42 (32.56%)	<0.0001
Renal failure	72 (6.69%)	15 (11.45%)	0.07
**Physical examination**			
Altered mental status	96(8.91%)	35 (26.72%)	<0.0001
Pulse ≥125 beats/min, n (%)	108 (10.03%)	8 (6.11%)	0.21
Respiratory rate ≥30 breaths/min, n (%)	144 (13.37%)	31 (23.66%)	0.0035
Systolic blood pressure <90 mmHg, n (%)	46 (4.27%)	6 (4.58%)	0.82
Temperature ≥40°C, n (%)	4 (0.37%)	0 (0%)	>.99
**Laboratory and X-ray findings**			
Glucose ≥250 mg/dL, n (%)	91 (8.45%)	11 (8.40%)	>.99
Blood urea nitrogen >30 mg/dL, n (%)	270 (25.07%)	63 (48.09%)	<0.0001
Sodium <130 mmol/L, n (%)	69 (6.41%)	10 (7.63%)	0.57
Haematocrit<30%, n (%)	24 (2.23%)	8 (6.11%)	0.02
PaO_2_<60 mmHg, n (%)	458 (42.53%)	76 (58.02%)	0.0010
pH<7.35, n (%)	47 (4.36%)	9 (6.87%)	0.19
Pleural effusion	114 (10.58%)	9 (6.87%)	0.22
Bilateral/multilobar	233 (21.67%)	33 (25.38%)	0.37
**Previous antibiotic**	242 (22.51%)	27 (20.61%)	0.66
**Complications**			
ICU admission, n (%)	53 (4.92%)	1 (0.76%)	0.02
Need for IMV, n (%)	18 (1.67%)	0 (0%)	0.25
Shock, n (%)	44 (4.09%)	5 (3.82%)	>.99
**PSI class, n (%)**			<0.0001
I-III	589 (54.69%)	24 (18.32%)	
IV-V	488 (45.31%)	107 (81.68%)	
**PSI Score, mean (SD)**	88.23 (31.60)	117.35 (30.36)	<0.0001

Data are presented as n (%) or mean (SD). COPD: chronic obstructive pulmonary disease; CHF: congestive heart failure; CAD: coronary artery disease; CVD; cerebrovascular disease; ICU: intensive care unit; IMV: invasive mechanical ventilation; PSI: pneumonia severity index.

Age ≥80 years, congestive heart failure (CHF), dementia, respiratory rate (RR) ≥30 breaths/min and blood urea nitrogen (BUN) ≥30 mg/dL were identified as predictors of one-year mortality and all combined in a multivariate model (Table 2). When testing the validity of the model, results indicated that the beta regression coefficient of the prognostic index (PI) was found to be 0.96 (95% CI: 0.78, 1.13. The likelihood-ratio test was performed to contrast that the slope is 1, yielding a non-statistically significant result (p = 0.586). In order to develop a one-year mortality CAP severity index, now called the one-year CAPSI (community-acquired pneumonia severity index), a prediction score was developed, with dementia being weighted with 6 points, followed by age ≥80 scored with 4 points, BUN >30 mg/dL scored with 3 points, and CHF and RR ≥30 breaths/min both scored with 2 points. Consequently, dementia and age were considered major risk factors, while BUN, CHF and RR were considered minor risk factors. S2 Table shows the statistical measures of performance with different cut-offs of the one-year CAPSI.

**Table 2:** Multivariate survival analysis for one-year mortality in the derivation and validation cohorts, i.e. one-year CAPSI.

	Derivation cohort				Validation cohort		
Variables	Beta (s.e.)	HR (95% CI)	p-value	Weight	Beta (s.e.)	HR (95% CI)	p-value
**Age (years)**				4			
≥80 vs. <80	0.87 (0.20)	2.39 (1.63,3.50)	<0.001		0.74 (0.21)	2.10 (1.40,3.15)	0.0003
**CHF**				2			
Yes vs. No	0.57 (0.27)	1.78 (1.05,3)	0.0331		0.52 (0.24)	1.68 (1.04,2.72)	0.03
**Dementia**				6			
Yes vs. No	1.20 (0.20)	3.33 (2.25,4.91)	<0.001		1.38 (0.21)	3.98 (2.64,5.99)	<0.001
**RR (breaths/min)**				2			
≥30 vs. <30	0.49 (0.21)	1.63 (1.09,2.45)	0.018		0.49 (0.21)	1.63 (1.08,2.47)	0.02
**BUN (mg/dL)**				3			
>30 vs. ≤30	0.66 (0.18)	1.93 (1.35,2.75)	<0.0003		0.44 (0.19)	1.55 (1.07,2.24)	0.02
C-index (s.e)	0.73 (0.024)				0.75 (0.025)		

Beta (s.e): beta regression coefficient with standard error; HR: hazard ratio; CI: confidence interval. CHF: congestive heart failure; RR: respiratory rate; BUN: blood urea nitrogen; C-index: concordance index with standard error for the one-year CAPSI as continuous variable.

**Fig 1:**
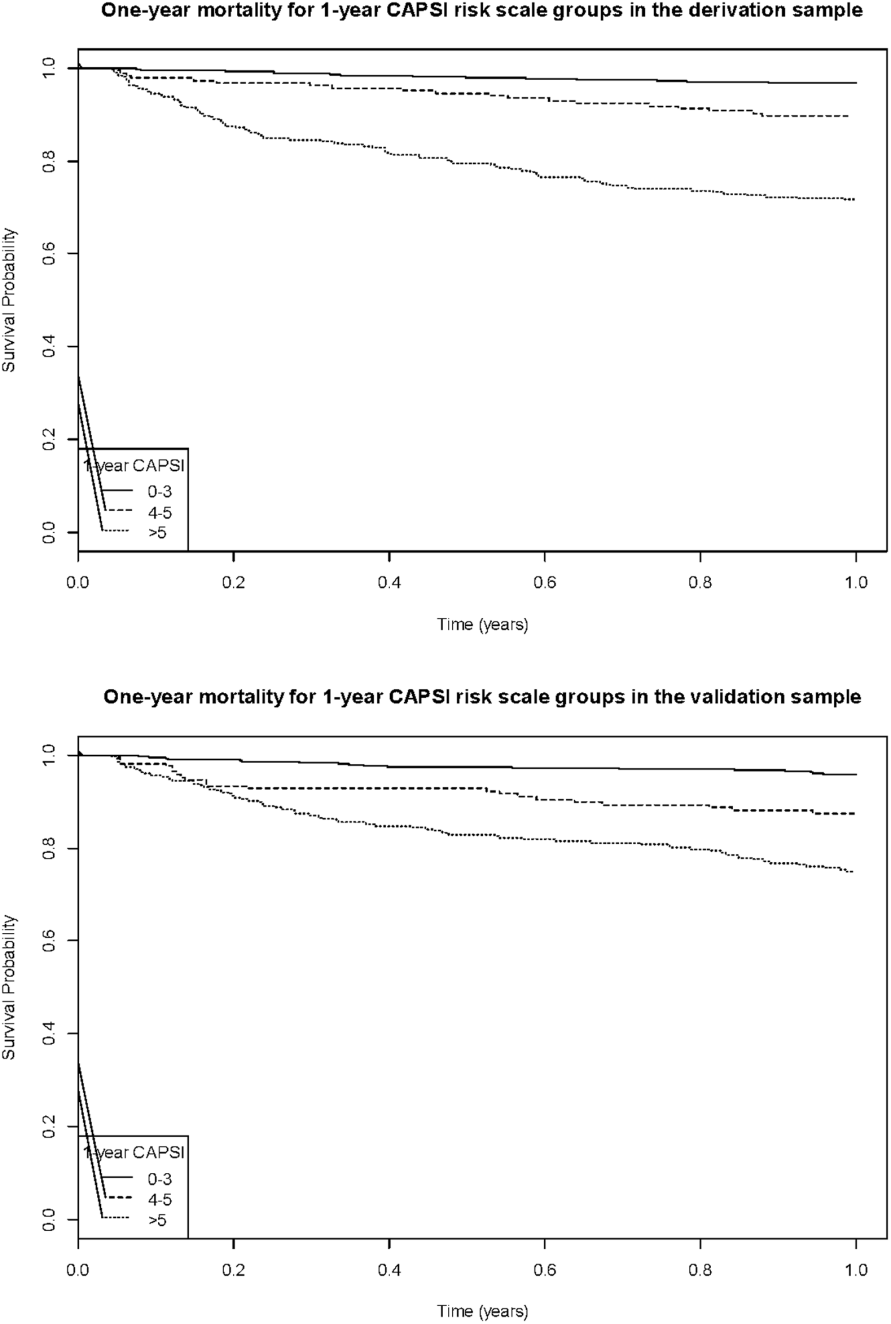
Kaplan-Meier survival curve for the derivation and validation cohorts, stratified by one-year CAPSI risk groups.

**Fig 2:**
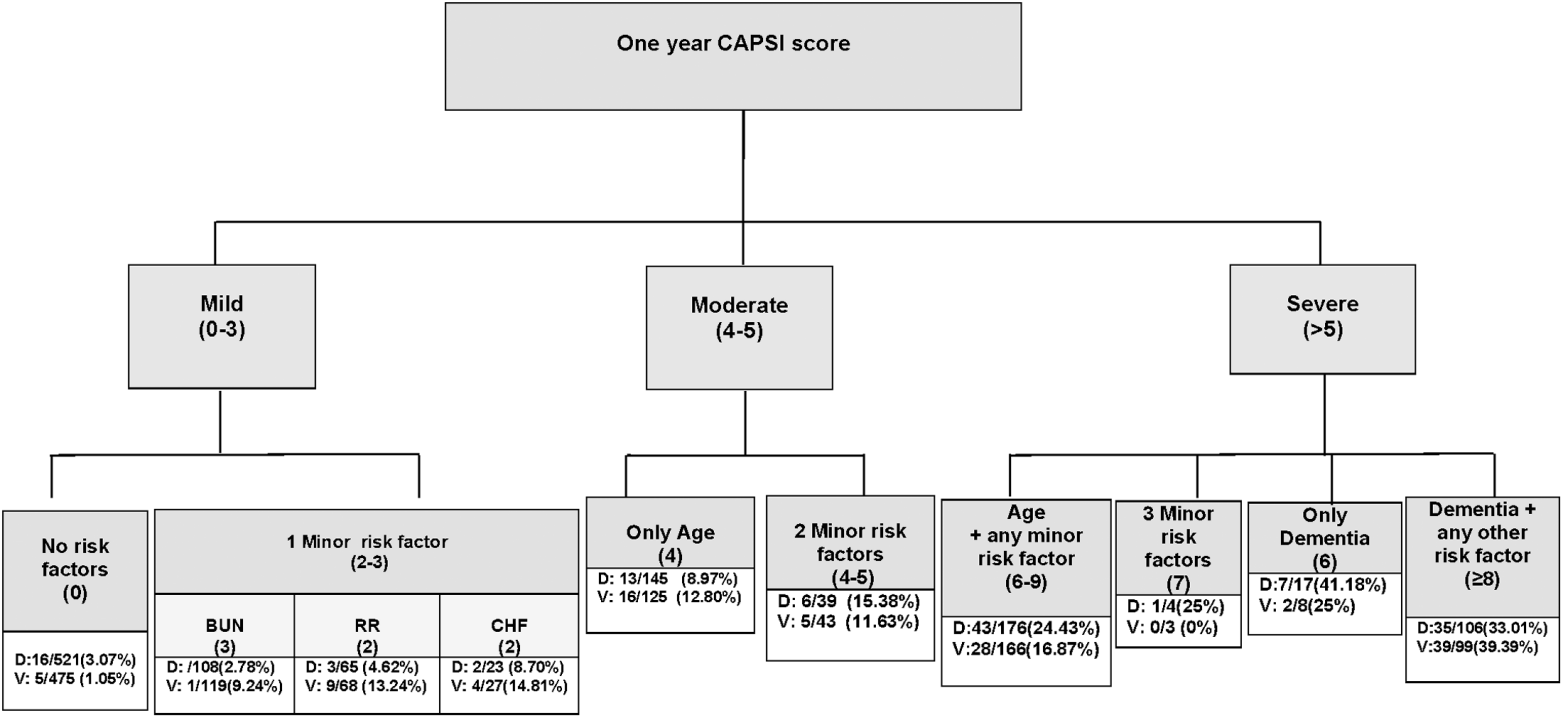
Risk-score distribution diagram according to the one-year CAPSI score risk groups. Minor risk factors: Chronic Heart Failure (CHF), Respiratory Rate (RR), Blood Urea Nitrogen (BUN). 2 minor risk factors: any pairwise combination of the minor risk factors. 3 minor risk factors: number of patients with the three minor risk factors. Age + any minor risk factor: age in combination with one, two or three minor factors. Dementia + any other risk factor: Dementia combined with other risk factor (age, one, two or three minor risk factors). D: number of deaths / number of patients at risk in the derivation sample. V: number of deaths / number of patients at risk in the validation sample.

For each unit increase in the score, the one-year mortality risk increased by 24% (1.24 [1.19, 1.28], HR [95% CI]). The one-year CAPSI was then categorized into three risk groups: mild, moderate, and severe risk, according to the one-year CAPSI risk score (Table 3). One-year mortality risk ranged from 3.35% in the lowest group to 28.38% in the highest group in the derivation cohort. Fig 1 shows Kaplan-Meier survival curves for the derivation and validation cohorts, stratified by the one-year CAPSI risk groups. Fig 2 shows the risk-score distribution according to the one-year CAPSI score risk groups.

**Table 3:** Multivariate survival analysis for one-year mortality in the derivation and validation cohorts by risk stratification groups.

	Derivation cohort			Validation cohort		
Variables	Dead/exposed	HR (95% CI)	p-value	Dead/exposed	HR (95% CI)	p-value
One-year CAPSI*	-	1.24 (1.19, 1.28)	<0.001		1.21 (1.17, 1.26)	<0.001
**One-year CAPSI**						
0–3	24/717 (3.35%)	Reference		29/689 (4.21%)	Reference	
4–5	19/184 (10.33%)	3.19 (1.75, 5.83)	0.0002	21/168 (12.5%)	3.03 (1.73, 5.32)	<0.001
>5	86/303 (28.38%)	9.91 (6.30, 15.59)	<0.001	69/276 (25%)	5.95 (3.86, 9.19)	<0.001
C-index (s.e)	0.72 (0.021)			0.70 (0.023)		
GND test	0.98			0.93		

HR: hazard ratio; CI: confidence interval. C-index: concordance index with standard error for the one-year CAPSI as categorical variable; GND test: Greenwood-Nam-D'Agostino calibration test for the one-year CAPSI as categorical variable.

*Hazard risk of each increase in one unit in the one-year CAPSI.

One-year CAPSI showed the best predictive accuracy in the derivation cohort with a C-index of 0.76, followed by PSI with a C-index of 0.73 (p = 0.18), CURB65 0.69 (p = 0.009) and SCAP 0.70 (p = 0.025). Likewise, one-year CAPSI showed the best predictive accuracy in the validation cohort with a C-index of 0.77, followed by PSI with a C-index of 0.75 (p = 0.39), CURB65 0.71 (p = 0.03), and SCAP 0.70 (p = 0.01). The p values refer to statistical differences between one-year CAPSI C-index with other prediction scores (Table 4).

**Table 4:** Predictive accuracy and goodness of fit for one-year mortality of continuous risk scores.

	Derivation			Validation		
	C-index (s.e)	AIC	R^2^	C-index (s.e)	AIC	R^2^
**PSI**	0.73 (0.024)	1750.74	7.6%	0.75 (0.025)	1556.01	9.1%
**CURB65**	0.69 (0.024)	1782.87	5.2%	0.71 (0.025)	1598.38	5.7%
**SCAP**	0.70 (0.024)	1784.05	5.1%	0.70 (0.025)	1608.79	4.8%
**One-year CAPSI**	0.76 (0.024)	1707.43	8.8%	0.77 (0.025)	1562.67	8.5%

C-index: concordance index with standard error for the one-year CAPSI as continuous variable; AIC: Akaike information criterion; R^2^: R-square; PSI: pneumonia severity index; CURB65: confusion, urea, respiratory rate, blood pressure, age>65. SCAP: severe community-acquired pneumonia; one-year CAPSI: one-year community-acquired severity index.

## Discussion

This study shows that long-term mortality in hospitalized patients with CAP is high. An easy-to-use score with five variables can help physicians identify those patients with CAP at high risk of death within one year of an index admission. A weighted score, called the one-year CAPSI, constructed by age ≥80, CHF, dementia, BUN >30 mg/dL and RR ≥30 breaths/min can predict one-year mortality with high predictive accuracy. Indeed, one-year CAPSI showed significantly better predictive accuracy than CURB65 and SCAP.

Dementia was the best predictor of one-year mortality in hospitalized patients with CAP. Dementia is defined as chronic cognitive impairment; its origin is usually due to cerebrovascular disease, which is closely related to inflammation [15,16]. Patients with CAP suffer an inflammatory storm during the acute episode, which could lead to worsening of a previously existing chronic inflammation process. Chronic persistent inflammation after an acute episode with CAP could be responsible for the high long-term mortality in these patients. There are other variables usually related to dementia in clinical practice, such as being a nursing home resident and having poor functional status, with the former included in the HCAP concept [17]. Patients with HCAP are known to have a worse prognosis, mostly due to poor functional status and treatment restrictions [18]. Cecere et al. [19] also described high long-term mortality in patients with HCAP, even higher than in patients with CAP. However, HCAP and previous functional status could not be assessed in the present study.

The second most powerful predictor of one-year mortality was age ≥80 years. Sir William Osler said that “Pneumonia may well be called the friend of the aged”. However, doubts have arisen in recent decades about pneumonia being limited to an acute episode. Due to the growing elderly population, the number of patients being admitted to hospital has notably increased. Moreover, these patients usually have more comorbidities and poor functional status. In a recent study, in which CAP patients were compared with a control group, the lowest absolute rate difference for mortality was observed among patients <25 years old, while patients >80 years old had the highest absolute rate difference [20]. However, the age at which risk increases is controversial, ranging from 50 years proposed by Hedlund et al. [21] to 70 years proposed by Sligl et al. [16], or 80 years as proposed in the one-year CAPSI. Though we assumed a practical point of view with the criteria that clinicians are used to employ categorical variables, future models could consider the use of continuous variables if easiness of use and availability for clinicians is warrantied through, for example, the use of computer facilities.

The severity of illness has been shown to have an impact on long-term prognosis. Scores such as the PSI or CURB65, initially developed for short-term prognosis, have been also used to assess long-term mortality, with contradictory findings. All these scores include data from a physical examination and laboratory tests at the time the index CAP episode was diagnosed, with two variables that were also included in the one-year CAPSI: BUN >30 mg/dL was weighted with 3 points and RR ≥30 breaths/min with 2 points.

A few authors have tried to explain the relationship between mortality and high BUN levels. The neurohumoral response to arterial underfilling may be responsible for this association. This response involves the renin-angiotensin-aldosterone system, arginine vasopressin (AVP) and the sympathetic nervous system [22]. Flow-dependent urea reabsorption increases due to the increased reabsorption of proximal tubular sodium and water and decreasing distal fluid delivery [23]. Hypoperfusion states are common in other illnesses, such as, myocardial infarction, necrotizing pancreatitis and sepsis [24]. Critical illnesses usually are associated to persistent hypercatabolism that may result in decreased immune function and in a higher long-term mortality [25]. In addition, Beier et al. [26], suggested that elevations in BUN affect mortality independent of creatinine due to the extent of catabolism.

It is more difficult to explain how an acute parameter like RR ≥30 breaths/min was also associated with one-year mortality after hospitalization for CAP. A high RR reflects the severity of disease, and has been frequently associated with a poorer short-term prognosis in patients with CAP. Thus, the impact of a high RR may reflect the impact of severity of illness on long-term prognosis.

In the last decade, substantial evidence has accumulated concerning the association between cardiovascular diseases and pneumonia. Firstly, patients suffering from CAP present higher rates of cardiac complications during hospitalization for CAP, such as acute myocardial infarction, heart failure, and arrhythmia [27,28]. These findings were supported by Dong et al. [29] in a meta-analysis showing that acute respiratory infections were associated with a higher rate of acute coronary syndromes. Secondly, it has been postulated that pneumonia increases the risk of developing new-onset heart failure and other cardiovascular diseases in late follow-up [30]. Thirdly, patients with CAP who experience intrahospital cardiac complications have greater long-term mortality [31]. CHF is included in the one-year CAPSI, and in fact has considerable impact on long-term mortality. Pneumonia leads to an inflammatory storm and factors such as systemic inflammation, coronary artery inflammation, platelet activation and thrombosis, endothelial dysfunction, and the effects of CAP on the heart have been suggested as possible mechanisms for increased cardiovascular events following respiratory infections [32].. Pneumonia may therefore lead to a chronic state of inflammation, triggering the onset of new cardiovascular events or exacerbation of a previously present inflammatory state.

Identifying the best prognostic index in any disease is difficult. CURB65 and PSI have been recently assessed for long-term mortality in a six-year follow-up study in patients with CAP [33] with both PSI and CURB65 showing excellent predictive accuracy. However, other authors have demonstrated that PSI was the best for predicting long-term mortality [34,35,33]. A good prognostic score should be easily performed in clinical practice. In this sense, the one-year CAPSI is based on five easy to remember variables and does not require complex computer programs to be implemented. More recently, a new score to predict one-year mortality after pneumonia focused on elderly and with a retrospective design was developed [36]. Our one-year CAPSI score, instead, was based on a prospective study with a large sample size and was performed on all age and severity groups. Contrary to Putot et al. [36], this new score is constructed by clinical variables while biomarkers measures are not needed.

The one-year CAPSI can help us to better stratify these patients at high risk of dying at late follow-up. After an episode of pneumonia, patients are usually evaluated in the short-term, which is probably sufficient for some; however, there are a few patients who should be considered for closer monitoring at late follow-up. Thus, patients with no risk factors according to the one-year CAPSI could continue with current follow-up protocols. For those with one or two minor risk factors or age ≥80, closer monitoring should be recommended, such as re-evaluation 6 months after the initial episode, focusing on the development or worsening of cardiovascular diseases. However, as shown in the risk-score distribution diagram (Fig 2), the risk increases when CHF is present compared to other minor risk factors. Thus, patients with CHF should be carefully monitored for any clinical signs of deterioration. In addition, quarterly monitoring should be strongly recommended for patients in the severe group. Of course, there are factors that cannot be modified, such as age or acute parameters and similarly, little is known about dementia onset or progression. Nevertheless, physicians should pay particular attention to reducing vascular risk, and should consider new treatment strategies in patients at high risk of dying after an episode of pneumonia.

This study has also several limitations. Firstly, causes of death could not be obtained in any case, which could have added important information. Secondly, several variables such as tobacco use, albumin, or platelets could not be assessed. Thirdly, patients who had been discharged from any healthcare centre in the previous 14 days, HIV-positive patients and chronically immunosuppressed patients were excluded from the study; thus, results cannot be generalized to such individuals. Fourthly, the proportion of eligible but non-included patients is unknown. Fifthly, external validation is needed to demonstrate generalizability of the model. However, the TRIPOD statement relating to development of clinical prediction rules was adhered to in all cases. In addition, future external validation in another cohort is likely to be conducted in the near future. Finally, although the predictive ability of our model was good, some other factors from the admission and follow up not included in our score could improve this model.

## Conclusions

An easy-to-use score, one-year CAPSI, based on five parameters can predict one-year mortality with high predictive accuracy, better than widely known severity scores. Recognizing these five variables may be useful for identifying patients with a high probability of dying after an episode of CAP. Future research should be conducted to clarify the impact of inflammation on CAP prognosis.

## Supporting information

S1 Table Baseline characteristics of study participants in the derivation and validation cohorts. (DOCX)

S2 Table Statistical measures of performance with different cut-offs for one-year CAPSI. (DOCX)
