# Supplementary material for: Predicting 1-year mortality after hospitalization for community-acquired pneumonia
Source: PLoS One. 2018 Feb 14;13(2):e0192750. doi: 10.1371/journal.pone.0192750 (PMC5812619; doi:10.1371/journal.pone.0192750)
Supplement: S1 Table — (DOCX) [file pone.0192750.s001.docx]

**S1 Table. Baseline characteristics of study participants in the derivation and validation cohorts.**

|  | **Derivation**  **(n=1208)** | **Validation**  **(n=1143)** |
| --- | --- | --- |
| **Age, mean (SD)** | 69.68 (16.51) | 69.94 (16.66) |
| **Sex, n (%)** |  |  |
| Male | 788 (65.23%) | 752 (65.79%) |
| Female | 420 (34.77%) | 391 (34.21%) |
| **Alcohol consumption (yes), n (%)** | 63 (5.23%) | 52 (4.59%) |
| **Comorbidities, n (%)** |  |  |
| Diabetes mellitus | 191 (15.92%) | 171 (15.13%) |
| COPD | 278 (23.11%) | 335 (29.54%) |
| Cancer | 76 (6.29%) | 54 (4.72%) |
| CHF | 81 (6.71%) | 99 (8.66%) |
| CAD | 119 (9.88%) | 105 (90.74%) |
| CVD | 105 (8.69%) | 94 (8.22%) |
| Dementia | 123 (10.18%) | 107 (9.36%) |
| Renal failure | 87 (7.20%) | 89 (7.79%) |
| **Physical examination** |  |  |
| Altered mental status | 131 (10.84%) | 116 (10.15%) |
| Pulse ≥125 beats/min, n (%) | 116 (9.60%) | 117 (10.24%) |
| Respiratory rate ≥30 breaths/min, n (%) | 175 (14.49%) | 179 (15.66%) |
| Systolic blood pressure <90 mmHg, n (%) | 52 (4.30%) | 45 (3.94%) |
| Temperature ≥40°C, n (%) | 4 (0.33%) | 7 (0.61%) |
| **Laboratory and X-ray findings** |  |  |
| Glucose ≥250 mg/dL, n (%) | 102 (8.44%) | 95 (8.31%) |
| Blood urea nitrogen >30 mg/dL, n (%) | 333 (27.57%) | 328 (28.70%) |
| Sodium <130 mmol/L, n (%) | 79 (6.54%) | 73 (6.39%) |
| Haematocrit<30%, n (%) | 32 (2.65%) | 25 (2.19%) |
| PaO_2_<60 mmHg, n (%) | 534 (44.21%) | 490 (42.87%) |
| pH<7.35, n (%) | 56 (4.64%) | 48 (4.20%) |
| Pleural effusion | 123 (10.18%) | 112 (9.80%) |
| Bilateral/multilobar | 266 (22.07%) | 264 (23.14%) |
| **Previous antibiotic** | 269 (22.31%) | 263 (23.09%) |
| **Complications** |  |  |
| ICU admission, n (%) | 54 (4.47%) | 52 (4.55%) |
| Need for IMV, n (%) | 18 (1.49%) | 14 (1.22%) |
| Shock, n (%) | 49 (4.06%) | 42 (3.67%) |
| **PSI class, n (%)** |  |  |
| I-III | 613 (50.75%) | 544 (47.59%) |
| IV-V | 595 (49.25%) | 599 (52.41%) |
| **PSI Score, mean (SD)** | 91.39 (32.73) | 91.58 (31.65) |

Data are presented as n (%) or mean (SD). COPD: chronic obstructive pulmonary disease; CHF: congestive heart failure; CAD: coronary artery disease; CVD; cerebrovascular disease; ICU: intensive care unit; IMV: invasive mechanical ventilation; PSI: pneumonia severity index.
