# Supplementary material for: Predicting 1-year mortality after hospitalization for community-acquired pneumonia
Source: PLoS One. 2018 Feb 14;13(2):e0192750. doi: 10.1371/journal.pone.0192750 (PMC5812619; doi:10.1371/journal.pone.0192750)
Supplement: S2 Table — (DOCX) [file pone.0192750.s002.docx]

**S2 Table. Statistical measures of performance with different cut-offs for one-year CAPSI.**

|  | **Sensitivity (%)**  **(95% CI)** | **Specificity (%)**  **(95% CI)** | **PPV (%)**  **(95% CI)** | **NPV (%)**  **(95% CI)** | **Accuracy (%)**  **(95% CI)** |
| --- | --- | --- | --- | --- | --- |
| **Derivation** |  |  |  |  |  |
| ≥4 | 81.40 (73.59-87.70) | 64.47 (61.52-67.33) | 21.56 (17.99-25.48) | 96.65 (95.06-97.84) | 66.28 (63.53-68.95) |
| ≥6 | 95.23 (93.63-96.52) | 78.41 (75.97-80.70) | 66.67 (57.83-74.72) | 79.81 (77.29-82.18) | 28.38 (23.37-33.82) |
| ≥8 | 39.53 (31.04-48.52) | 90.70 (88.80-92.37) | 33.77 (26.29-41.91) | 92.59 (90.84-94.10) | 85.22 (83.08-87.17) |
| **Validation** |  |  |  |  |  |
| ≥4 | 75.63 (66.91-83.03) | 65.09 (62.06-68.02) | 20.27 (16.63-24-32) | 95.79 (94.01-97.16) | 66.20 (63.36-68.95) |
| ≥6 | 57.98 (48.59-66.97) | 79.59 (76.97-82.03) | 25 (20-30.54) | 94.17 (92.38-95.64) | 77.32 (74.77-79.73) |
| ≥8 | 40.34 (31.45-49.72) | 90.83 (88.88-92.53) | 34.04 (26.28-42.49) | 92.84 (91.06-94.37) | 85.53 (83.3487.52) |

NPV: negative predictive value; PPV: positive predictive value.
